# THE ASSOCIATIONS BETWEEN SOCIAL NETWORKS, CARE NETWORKS, AND WELL-BEING COMPARED IN DIFFERENT TIME PERIODS

**DOI:** 10.1093/geroni/igae098.0119

**Published:** 2024-12-31

**Authors:** Marjolein Broese Van Groenou, Silvia Klokgieters, Jens Abbing, Joukje Swinkels

**Affiliations:** Vrije Universiteit Amsterdam, Amsterdam, Noord-Holland, Netherlands; Vrije Universiteit Amsterdam, Amsterdam, Noord-Holland, Netherlands; Vrije Universiteit Amsterdam, Amsterdam, Noord-Holland, Netherlands; .VU Amsterdam, Amsterdam, Noord-Holland, Netherlands

## Abstract

Objectives A well-functioning LTC network is important for the psychological well-being of older CRs. Yet, the use of formal and informal care depends on the allocation of LTC which may vary over historic time. This raises the question whether the links between social networks, care networks and psychological wellbeing differ between various LTC policy contexts in the Netherlands. Methods Data are from 65-plus in four waves of the LASA study that are ten years apart: 1992 (N=1794), 2002 (N=1284), 2012 (N=1183), 2022 (1011). Five care network types were considered (no care, partner care, informal, formal and privately paid care). Per wave regression analyses are used to analyze the associations between social networks, care networks and depressive symptoms, and effects are compared across waves using multigroup analysis. Results Preliminary results show that the prevalence of care network types differ over time, with a decrease in partner and informal care types and stability in formal and privately paid care types. Many of the effects appeared stable over time: in all waves social network composition contributed to informal care use; and partner care reduced depressive symptoms where formal care increased this. Care network types that fostered relatedness and sufficient care were important for wellbeing in all waves. Discussion Although the four waves represent varying LTC contexts, the findings support the robustness of the associations between social networks, care networks and wellbeing. Moreover, those who lack a partner and a social network risk dependency on formal care resulting in lower wellbeing in all times.

